# Editorial: ADME of drugs to treat infectious diseases

**DOI:** 10.3389/fphar.2026.1896123

**Published:** 2026-07-07

**Authors:** Cyprian Onyeji, Zhihao Liu, Caisheng Wu

**Affiliations:** 1 Department of Pharmaceutical and Medicinal Chemistry, Faculty of Pharmaceutical Sciences, University of Nigeria, Nsukka, Nigeria; 2 Department of Nutrition and Food Science, University of Maryland, College Park, MD, United States; 3 School of Pharmaceutical Sciences, Xiamen University, Xiamen, Fujian, China

**Keywords:** ADME, algorithm development, basic research, clinical research, infectious diseases

## Background

Infectious diseases are pathogen-induced illnesses transmissible through various means among organisms. Owing to their epidemic nature, these diseases are highly prone to large-scale outbreaks within specific regions, posing severe threats to public health and socioeconomic development (Chen et al., 2025; Escudero-Pérez et al., 2023; Yadav and Pandey, 2022). The treatment of infectious diseases relies on therapeutic agents that directly target pathogens to block their replication and proliferation, while minimizing collateral damage to normal tissues and cells (Almeida Furquim de Camargo et al., 2023; He et al., 2021; Kunkel and McHugh, 2024). However, the therapeutic successes of these anti-infective agents do not only depend on their intrinsic potencies but it is also highly influenced by how they are processed within the human body.

This highlights the importance of Absorption, Distribution, Metabolism, and Excretion (ADME), a set of pharmacokinetic processes that not only determine systemic drug exposure but also govern target-site drug concentrations, thereby serving as major determinants of therapeutic outcomes in infectious diseases.

Importantly, the optimal ADME characteristics of anti-infective agents are highly dependent on the site of infection, the causative pathogen, and the intended route of administration. For example, antibiotics used for the treatment of urinary tract infections often benefit from low liver metabolism rate, high urine drug concentration, glomerular filtration, and hydrophilicity, allowing therapeutically effective concentrations to be achieved in urine. In contrast, agents intended for deep-seated tissue, intracellular, or bone infections generally require sufficient lipophilicity and favorable tissue distribution properties to penetrate biological barriers and reach infection sites. Likewise, intravenously administered anti-infective agents are primarily optimized for systemic exposure, rapid therapeutic attainment, and predictable pharmacokinetic behavior, whereas orally administered drugs must additionally possess adequate gastrointestinal absorption and physicochemical properties that support oral bioavailability, frequently aligning with established drug-likeness principles such as Lipinski’s Rule of Five (Lipinski et al., 2001). Therefore, ADME considerations in anti-infective drug development extend beyond conventional pharmacokinetic evaluation and represent critical determinants of target-site drug exposure, therapeutic efficacy, and clinical success.

Absorption, the process by which a drug gets into systemic circulation is followed by bioavailability which is a measure of the rate and extent to which they reach the sites of action. Distribution, which involves the transfer of a drug from systemic circulation into biological fluids and tissues is also an important factor in determining effective treatment of infectious diseases since satisfactory drug penetration into the infected site is a requirement. Drug distribution characteristics govern accumulation at the infected site as well as potential adverse effects on normal tissues (Carvalho et al., 2020; Ekpenyong et al., 2020; Sandker et al., 2022). Together, these factors influence whether intervention occurs before pathogen replication enters the plateau phase (Husain et al., 2025; Tanimura et al., 2003; Yang et al., 2024). Drug Metabolism, a biochemical transformation of drugs, has a number of consequences including activation of prodrugs, inactivation of active compounds, and generation of toxic metabolites from the drugs. Inter-individual variation in drug metabolism contributes to differences in drug efficacy, and toxicity (Hafner et al., 1998; Poornachandran et al., 2026; Tanigawara et al., 1995). Drug disposition and also be markedly influenced by alterations in Excretion. Collectively, these ADME investigations enable an integrated evaluation of the clinical translational potential of anti-infective therapeutic agents.

A total of 14 ADME-related studies on the treatment of infectious diseases were included in this Research Topic, which can be systematically classified into three categories: clinical research, basic/non-clinical research, and tool and algorithm development. Together, these contributions, summarized in Table 1, fully underpin the construction of an ADME research framework for anti-infective therapeutics.

**TABLE 1:** Summary of ADME-based research on anti-infective therapeutic agents.

Category	Focus area	Key methods/Approaches	Authors	Major findings/Contributions
Clinical research	Dose adjustment, drug–drug interactions, PK/PD optimization, special populations	Therapeutic drug monitoring (TDM), population pharmacokinetic (PopPK) modeling, clinical trials, systematic review	Wang H et al.; Zhou L et al.; Han M et al.; Wu J et al.; Zhang P et al.; Riese T et al.	Established how CRRT, ECMO, inflammation, and genetic polymorphisms affect drug exposure and clearance; underlined importance of TDM and individualized dosing in critically ill patients
Basic/Non-clinical research	Mechanistic investigation of ADME processes such as transporters, metabolism, tissue distribution, metabolomics	PBPK modeling, animal models, cell studies, microdialysis, metabolomics (^1^H-NMR), molecular pharmacokinetics	Wang F et al.; Santos VV et al.; Zuo Y et al.; Wang C et al.; Omotayo OP et al.	Ascertained strategic mechanisms such as lysosomal drug entrapment, drug-transporter interactions, toxicity mediated by transporter proteins, tissue drug penetration, drug-induced metabolic abnormalities
Tool & algorithm development	Simulation, evidence-based integration and prediction, clinical decision-support application, evaluation of the pharmacokinetic profiles	PBPK/mPBPK modeling, *in silico* simulation, web-based platforms, PK/PD modeling, clinical decision-support tools	Visintainer R et al.; Zhang S et al.	Established modelling platforms for simulating drug disposition (e.g., anti-TB drugs, remdesivir, fluconazole); enabled dose optimization through PK evaluation

### Clinical research

Clinical studies enroll patients or healthy volunteers to examine various aspects of pharmacotherapy. These studies focus on pharmacokinetics in special populations, dose optimization, drug-drug interactions, and individualized dosing, thereby providing direct evidence for safe and effective clinical pharmacotherapy. Wang et al. established a population pharmacokinetic model of voriconazole in patients with COVID-19-associated pulmonary aspergillosis. They identified that covariates such as continuous renal replacement therapy (CRRT) and inflammatory biomarkers significantly affect drug clearance, and proposed personalized dosing recommendations. Their findings emphasized the relevance of therapeutic drug monitoring (TDM) in patients with fungal co-infections. This work contributes to the expanding field of personalized pharmacotherapy of anti-infective agents. Further studies on voriconazole by Zhou et al. revealed the advantages of intravenous voriconazole administration in patients receiving CRRT. It was noted that the injectable formulation circumvents absorption variability and emphasizing the importance of model-guided dose adjustment.

In the study by Han et al. the interaction between the anti-influenza agent GP681 and itraconazole was evaluated in healthy subjects, confirming that CYP3A4 serves as the principal metabolizing enzyme and that genetic polymorphisms can alter metabolic efficiency. Itraconazole is a potent CYP3A4 inhibitor. This finding is clinically significant because patients receiving the antiviral therapy may often concurrently be administered with medications that inhibit CYP3A4 activity, thereby influencing drug treatment outcome. Further studies on itraconazole were done by Wu et al. who investigated the influence of itraconazole on the pharmacokinetics of ZX-7101A. The results revealed that the systemic exposure of ZX-7101A was lower following co-administration with itraconazole than after single-agent dosing. Although itraconazole is commonly recognized as a CYP3A4 inhibitor, it is also known to have effects on membrane transport proteins and these may also alter the pharmacokinetics of co-administered agents, resulting in reduced systemic exposure. Thus, dose optimization is required during multidrug therapy.

Zhang et al. conducted a systematic review of population pharmacokinetic studies of imipenem and integrated the available models. They identified creatinine clearance as the primary covariate and incorporated other clinical renal function indicators as key covariates for patient model construction, thus providing an important foundation for individualized dosing. Their study is a useful contribution to precision antimicrobial therapy, especially in critically ill patients where pharmacokinetics of imipenem can extensively vary. Riese et al. retrospectively analyzed the effects of extracorporeal membrane oxygenation (ECMO) and CRRT on plasma piperacillin (PIP) concentrations. They found that 45% of initial piperacillin concentrations in critically ill patients exceeded the recommended therapeutic range, and that plasma piperacillin levels surpassed the therapeutic window in patients receiving concurrent ECMO support. This study underscores the necessity of therapeutic drug monitoring (TDM) in the management of severe infections.

### Basic/non-clinical research

Basic/non-clinical research employs animal, cellular, and molecular models to elucidate the intrinsic mechanisms underlying drug absorption, distribution, metabolism, and excretion (ADME), thereby providing a theoretical foundation for clinical optimization strategies. Wang et al. conducted pharmacokinetic studies of tetrandrine (TET), established a physiologically based pharmacokinetic (PBPK) model in rats and dogs, and validated the model in humans. The study confirmed that the liver is the primary metabolic organ for TET and that lysosomal trapping represents the key determinant of its tissue distribution profile. This helps explain the characteristic tissue distribution profile of tetrandrine which might contribute to effectiveness of the drug in pulmonary and intracellular infectious diseases.

Santos et al. employed microdialysis combined with population modeling to compare the tissue penetration of amphotericin B (AmB) between healthy rats and those infected with *Candida* albicans. The results of this study suggest that the pharmacokinetics and tissue penetration of the drug were not altered despite the inflammatory and physiological changes associated with systemic *Candida* infection. Zuo et al. conducted a systematic analysis of the altered expression of renal drug transporters in renal cell carcinoma Their work provides an essential insight into how renal cell carcinoma is associated with substantial alterations in the expression and activity of transporters which may remarkably affect the *in vivo* disposition of drugs in cancer patients. Wang et al. demonstrated from the perspective of molecular pharmacokinetics that JBP485 attenuates the nephrotoxicity and oxidative stress induced by aristolochic acid I through inhibition of renal organic anion transporters (OATs), thereby reducing renal accumulation of aristolochic acid I. The study is important because, it demonstrates that alteration of drug-transporter interactions can impact on tissue distribution of toxic compounds. The work by Omotayo et al. applied ^1^H-nuclear magnetic resonance (^1^H-NMR) metabolomics to investigate how anti-tuberculosis (anti-TB) combination therapy alters metabolism in mice.

Metabolomics involves a complete analysis of small endogenous metabolites present in biological samples. The authors in this study used ^1^H-NMR spectroscopy to simultaneously analyse several metabolites. This enabled provision of metabolic changes which occurred and thereby establishing a research paradigm for distinguishing drug-induced metabolic abnormalities from infection-driven metabolic perturbations.

### Tool and algorithm design

Tool and algorithm development focuses on mathematical modeling, simulation platform construction, and evidence-based integration, providing standardized methodologies for rapid prediction, therapeutic regimen design, and efficacy evaluation. A study by Visintainer et al. developed a web-based minimal physiologically based pharmacokinetic (mPBPK) platform for studying anti-tuberculosis drugs in murine tuberculosis models. A minimal PBPK (mPBPK) model reduces PBPK model complexity while still retaining important features needed for prediction of drug behavior. In this work, the authors used the simulation tool to enable evaluation of the pharmacokinetic profiles of 11 anti-tuberculosis drugs.

Zhang et al. employed *in silico* pharmacokinetic modeling to simulate the pharmacokinetic characteristics of remdesivir and its metabolites in healthy individuals and patients with renal impairment. The developed mathematical model demonstrated excellent concordance with experimentally observed data. Furthermore, Zhang et al. constructed a population pharmacokinetic/pharmacodynamic (PK/PD) model to optimize fluconazole dosing regimens for patients with acute renal failure receiving CRRT. On this basis, a clinical decision-support application was further developed to facilitate individualized therapeutic management.

## Conclusion

In conclusion, this Editorial highlights the essential role of ADME in determining the therapeutic efficacy and safety of antimicrobial agents. The article reviewed fourteen ADME-related studies which were categorized into three major areas: clinical research, basic/non-clinical research, and tool/algorithm development. The three categories of studies are mutually complementary and collectively form a comprehensive research framework spanning fundamental drug research, clinical application, and tool design with algorithm development. This framework provides robust theoretical foundations and practical algorithmic tools for ADME research in the field of infectious diseases. Overall, the findings from these studies underline the importance of application of ADME in personalized therapy, therapeutic drug monitoring, metabolomics, drug transporter systems, and PBPK modeling in management of infectious diseases.

It is anticipated that the aforementioned studies will advance ADME research in infectious diseases, offer innovative perspectives for both basic investigations and clinical translation, and further promote the development of this important field.
